# Identification of Anisotropic Coefficients in the Non-Principal Axis Directions of Tubular Materials Using Hole Bulging Test

**DOI:** 10.3390/ma16134629

**Published:** 2023-06-27

**Authors:** Yanli Lin, Yifan Wang, Yibo Su, Junpeng Liu, Kelin Chen, Zhubin He

**Affiliations:** 1 School of Mechanical Engineering, Dalian University of Technology, Dalian 116024, China; linyanli@dlut.edu.cn (Y.L.); zxc812902250@163.com (Y.W.); suyibo2021@163.com (Y.S.); 935795196@mail.dlut.edu.cn (J.L.); kchen@dlut.edu.cn (K.C.); 2 State Key Laboratory of High-Performance Precision Manufacturing, Dalian 116024, China

**Keywords:** anisotropic coefficient, thin-walled tube, AA6061-O extruded tube, hole bulging test, parameter identification

## Abstract

We propose an experimental method to identify anisotropic coefficients in non-principal axis directions of thin-walled tubes. The method involves extracting specimens from the parent tubes and machining a hole in the axial center. The specimens are then inserted into a tube without a hole. The inner diameter of the specimen is theoretically equal to the outer diameter of the inner tube. The double-layer tube undergoes free bulging under internal pressure in our self-developed experimental equipment, with the hole on the specimen expanding simultaneously. The stress states around the hole are uniaxial, and the hole deformation can reflect the anisotropic plastic flow characteristics of the tube. Furthermore, based on the information obtained from the proposed experimental method, a hybrid numerical–experimental method was used to identify the anisotropic coefficients of tubes. Through FE simulations, the relationships between the thickness, stress, and strain states around the hole, the hole shape, and anisotropic coefficients of non-principal axis directions are revealed, and the factors that affect the hole deformation are analyzed. Finally, the hole bulging experiments and FE simulations of AA6061-O extruded tube were conducted, and modeled with Hill48 and calibrated by uniaxial tensile and hoop tensile tests. Its in-plane anisotropy coefficients in any direction are given for the first time which first increase and then decrease from 0° to 90°, reaching a maximum of 1.13 in 60° and a minimum of 0.69 in 0°. This work can provide the key experimental data for establishing an accurate anisotropic plastic constitutive model of thin-walled tubes.

## 1. Introduction

Tube hydroforming is an advanced forming technology for the production of hollow and variable cross-section complex parts which are widely used in the aerospace and auto industries [1,2,3,4]. However, tube hydroforming results are greatly affected by the loading path, and extremely prone to cracking or wrinkling [5,6,7]. The shapes of the formed parts are generally complex, and thin-walled tubes exhibit significant plastic anisotropy [8,9,10], making it difficult to accurately predict the reasonable loading paths through theoretical analysis and experiments [11]. Therefore, FE simulation is usually used to determine and optimize process parameters before experiments [12]. However, the accuracy of FE simulation largely depends on the accuracy of the constitutive model used to describe the material properties including the plastic flow, yielding, and hardening characteristics [13]. Hence, constructing an accurate plastic constitutive model for thin-walled tubes is crucial for achieving accurate FE simulation of tube hydroforming. The anisotropy coefficients are usually used to calibrate the plastic potential and yield function, which are important for accurately reflecting the plastic deformation. Therefore, the anisotropy coefficients of the thin-walled tube in any direction are very important for constructing its accurate plastic constitutive model [14].

However, it is impossible to measure the anisotropy coefficients in any direction through uniaxial tensile tests such as sheet metal for thin-walled tubes due to its closed structure along the hoop direction [15]. At present, only the anisotropy coefficients along the axial and hoop directions can be measured by the uniaxial tensile test and the hoop tensile test [16,17,18], respectively. Some researchers performed uniaxial tensile tests by flattening specimens which are cut in different directions of the tube [19]. However, this method would produce pre-strain or even rupture during the flattening process, which causes the test results to be inaccurate.

In order to obtain the anisotropy coefficients of thin-walled tubes in the non-principal axis directions, some scholars first calibrate the anisotropic plastic constitutive model of thin-walled tubes through some experimental data of tension-tension [20,21,22,23] or even tension-compression [24,25,26] stress states, and then reverse determine the anisotropy coefficient through the flow rule. However, the two kinds of experimental data can only be used to calibrate the coefficients related to normal stress in the plastic constitutive model, while the coefficients related to shear stress in the plastic constitutive model cannot be determined. Due to lack of experimental data to calibrate the coefficients related to shear stress components in the plastic constitutive model of tubes, some researchers [26,27] assume them to be isotropic. Then the anisotropic coefficients of tubular materials in any direction are inversely calculated by the flow rule. For example, Kuwabara et al. [14] assumed that the undetermined coefficients $\alpha_{7}$ and $\alpha_{8}$ in the Yld2000 yield criteria are 1. However, the yield criteria based on this assumption cannot correctly describe the anisotropic plastic flow characteristics in the non-principal axial directions, so the anisotropic coefficients of thin-walled tubes in any non-principal axis directions determined by this method are not accurate. 

To address the problem, Zhang et al. [25] proposed an experimental method for measuring the axial pure shear yield stress of thin-walled tubes. Based on the assumption that the coefficients $\alpha_{7}$ and $\alpha_{8}$ of the Yld2000 yield criteria are equal, $\alpha_{7}$ and $\alpha_{8}$ are determined by pure shear yield stress. Further, the anisotropy coefficients in any direction of thin-walled tubes can be determined inversely by the associated flow rule. However, the axial pure shear yield stress can only reflect the anisotropic yield characteristics of thin-walled tubes, and cannot accurately reflect the anisotropic plastic flow characteristics in the non-principal axis directions. The anisotropy coefficients of thin-walled tubes in the non-principal axis directions determined by this method are still inaccurate.

In summary, the anisotropy coefficients of thin-walled tubes in the non-principal axis directions are the key data for calibrating the plastic constitutive models. However, so far, there are few studies about it that have been publicly reported.

In this paper, in order to identify anisotropic coefficients in non-principal axis directions of thin-walled tubes, a new experimental method of the hole bulging test (HBT) is proposed, which is inspired by the hole expansion tests of sheets. The deformation characteristics at the hole periphery can reflect the anisotropic plastic flow characteristics of tubes. Based on this experiment, the anisotropy coefficients of thin-walled tubes in any non-principal axis direction are determined by a hybrid numerical–experimental method. The FE simulations and experiments are used to analyze the deformation characteristics and influencing factors of the hole periphery during the experiment, and the feasibility of the method is verified. Furthermore, the hole bulging experiments of aluminum alloy AA6061-O extruded tube are conducted, and its in-plane anisotropy coefficients in the non-principal axis directions are successfully obtained, which will provide the key experimental data for establishing an accurate anisotropic plastic constitutive model.

## 2. Hole Bulging Test (HBT) and Hybrid Numerical–Experimental Method

### 2.1. Test Procedure of Hole Bulging Test

The hole bulging test (HBT) is proposed to determine the anisotropy coefficients of tubular materials, the idea of which is inspired by the hole expansion test (HET) of sheet metal. HET is a widely used method to measure the forming properties of the edge quality [28,29,30,31]. In recent years, more than the formability evaluation, the HET is increasingly being used to validate the anisotropic plastic constitutive model of materials, including plastic anisotropy [30,31,32] due to the following reasons: (1) larger deformation can be achieved than a standard uniaxial tensile test due to compatibility with the surrounding materials; (2) the material is deformed under various stress states between uniaxial tension, plane strain tension, and equal biaxial tension from the hole edge and radially inland [29]. Furthermore, the deformation of the hole during the HET has been used to determine the anisotropy coefficients of sheet metal in any direction [33].

**Figure 1:**
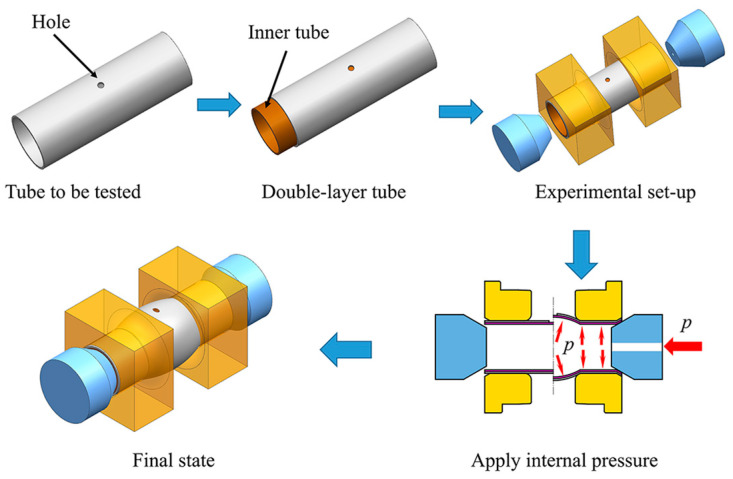
Schematic of the HBT.

**Figure 2:**
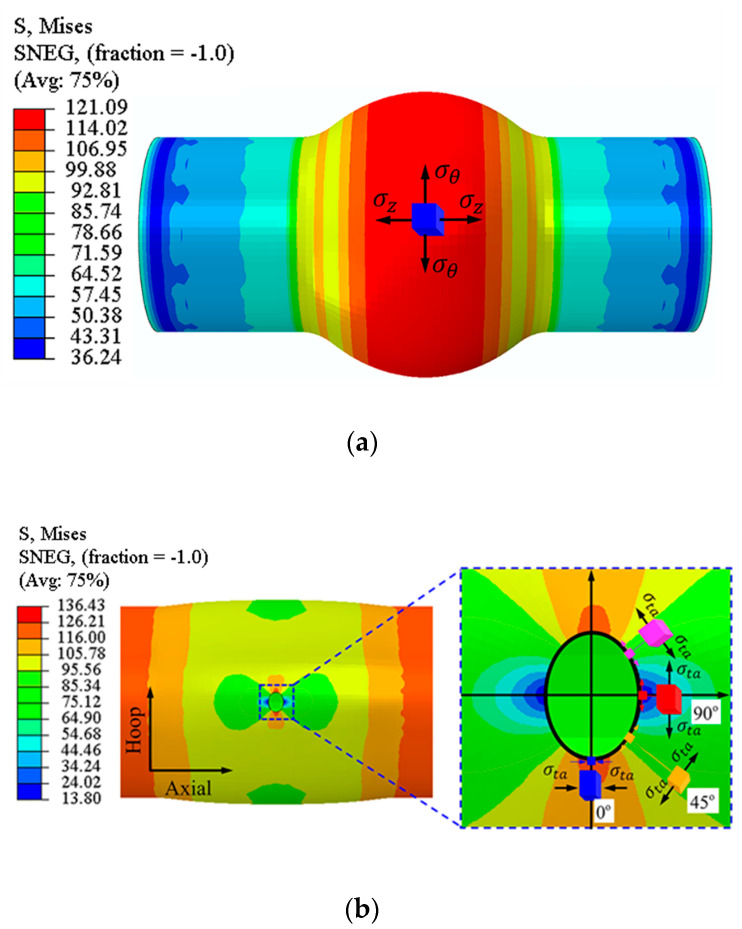
(**a**) Stress state of single-layer tube during free bulging, and (**b**) Stress state of the HBT.

For the HBT, the specimen is a tube with a hole in the axial center. Due to the fact that the tube is a circumferentially closed structure, how to deform the hole is completely different from how to expand the hole in the sheet metal. To solve this problem, the tubular specimen is inserted into a tube without a hole (inner tube), and the inner diameter of the tubular specimen is theoretically equal to the outer diameter of the inner tube. The double-layer tube undergoes free bulging under the internal pressure applied to the inner tube, and the hole on the specimen expands simultaneously. The schematic of the HBT is shown in Figure 1. The edge of the hole on the tubular specimen is free, so that it can deform freely and the stress state is uniaxial, which is similar to that of the hole expansion test of sheet metal but completely different from the stress state of the single-layer tube free bulging test (see Figure 2). The stress direction of any point at the hole periphery is along the tangent direction. Each tangent line can represent a direction in the tube plane (see Figure 2b). All directions in the tube plane are considered to be involved in the deformation of the hole periphery. From the stress–strain analysis of Section 4.2, the plastic deformation occurs in almost all directions of the hole periphery. Therefore, the deformation characteristics of the hole (such as the hole shape, the thickness of the hole periphery, etc.) can reflect the anisotropic plastic flow characteristics in any direction in the tube plane, which can be used to determine the anisotropy coefficients in the tube plane.

### 2.2. Identification of Anisotropy Coefficients by Hybrid Numerical–Experimental Method

**Figure 3:**
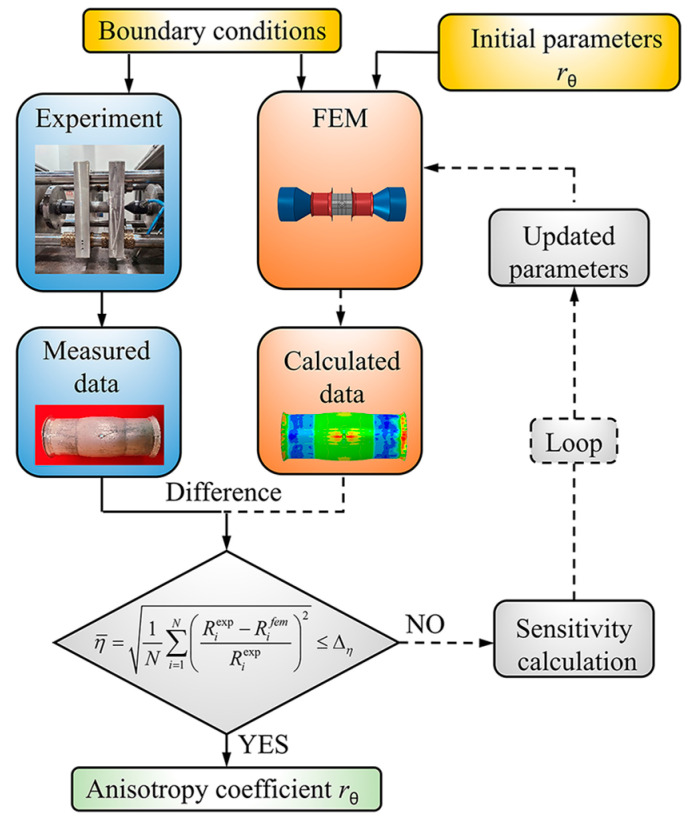
Flow-chart of hybrid numerical–experimental method.

The HET can directly obtain the anisotropy coefficients of the sheet metal in any direction by measuring the strain [33]. However, due to the particularity of the tube structure, the anisotropy coefficients of the tube cannot be directly obtained by the same method. Therefore, the hybrid numerical–experimental method shown in Figure 3 is proposed to determine the in-plane anisotropy coefficients of tubes.

The method is based on the results of the deformation characteristics of the hole in the HBT, and the simulation analysis is carried out by changing the undetermined parameters of the constitutive model. When the error between the results of FE simulation and experiments is reduced to an allowable range, the parameters of the constitutive model used in the simulation are the actual values of the material. The parameters of the constitutive model are generally calibrated by the in-plane anisotropy coefficient $r_{\theta }$, the subscript *θ* represents the angle with respect to the axial direction. The anisotropy coefficients in the principal axis directions, *r*_0_ and *r*_90_, can be measured by experiments, while the anisotropy coefficients in other directions such as *r*_45_ cannot be obtained by experiments which are the undetermined parameters of the constitutive model and need to be determined through the hybrid numerical–experimental method, as shown in Figure 3. After the anisotropy coefficients of these specific non-principal directions are determined, the constitutive model can be determined, and further, the anisotropy coefficients of any other non-principal directions can be determined by the constitutive model.

The deformation characteristics of the hole in the HBT include the hole shape and the thickness at the hole periphery. The hole shape can be characterized by the long axis of the deformed hole in this study (hereinafter referred to as the aperture). 

The accurate determination of the anisotropy coefficients needs a reasonable cost function. The cost function is defined as the root mean square error (RMSE) between the results of FE simulations and experiments:

(1) $$\bar{\eta }=\sqrt{\frac{1}{N}{\sum_{i=1}^{N}\left(\frac{R_{i}^{exp}-R_{i}^{fem}}{R_{i}^{exp}}\right)}^{2}}$$

where *N* is the number of experiments, $R_{i}^{exp}$ and $R_{i}^{fem}$ are the values of the iterative metric in the results of experiment and simulation, respectively.

The number of non-principal anisotropy coefficients to be determined is related to the constitutive model selected. When there is only one coefficient to be determined, a reasonable RMSE threshold $\Delta_{\eta }$ is selected. Firstly, the bisection iteration is carried out to quickly reduce the iteration interval. When $\bar{\eta }\leq \Delta_{\eta }$ terminates the iteration, the $r_{\theta }$ is the optimal result. If the iteration step size $\Delta r_{\theta }<0.05$ and the RMSE $\bar{\eta }>\Delta_{\eta }$ appears in the iteration process of the bisection iteration, the fixed step search is carried out in the interval. The search step size of the fixed step search is $\Delta r_{\theta }=0.01$. If the iteration error increases as the iterative step size increases or decreases, it indicates that the iteration error has reached the minimum value. Therefore, the $r_{\theta }$ at this point can be considered the optimal result. When the number of non-principal anisotropy coefficients to be determined is more than one, the grid search method can be used to determine the optimal solution of each non-principal anisotropy coefficient.

### 2.3. Experiment Equipment of Hole Bulging Test

**Figure 4:**
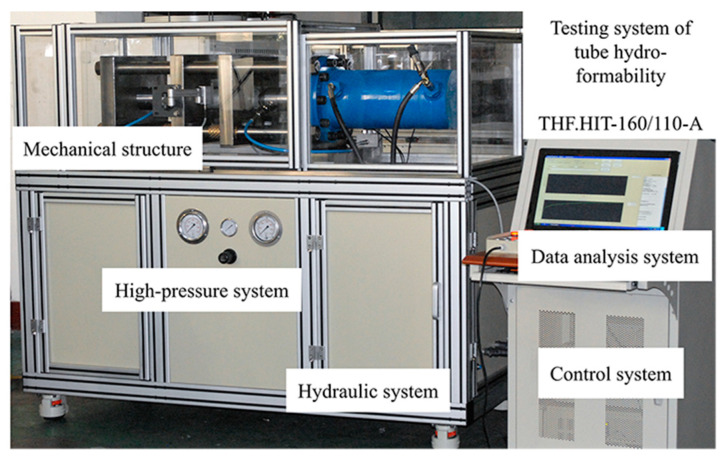
Tube free bulging test equipment.

The home-developed THF.HIT-160/110-A tube mechanical properties test equipment was used for the HBT, as shown in Figure 4. The equipment is composed of mechanical structure, high-pressure system, hydraulic system, control system, and data analysis system. The free bulging test of the tube can be conducted on it, through which the mechanical properties of tubes such as stress–strain component curves and hardening curves can be obtained. A maximum axial force of 50 t and a maximum working pressure of 160 MPa can be provided by the equipment.

### 2.4. Advantages and Disadvantages of the HBT

The advantages of the HBT include the following: (1) The specimen is a tube with a hole, which is directly extracted from the parent tube without changing the curvature shape of the tested tube and introducing additional strain hardening; (2) The edge of the hole is a free boundary and is subjected to uniaxial stress state, stress state is simple; (3) The experimental process only requires the application of internal pressure, which is simple and does not require a complex feedback control system; (4) The experiment can be conducted on free bulging test equipment without the need for additional development of new devices.

Although this experiment has the above advantages, it also has some disadvantages, such as: (1) the influencing factors during the experimental process are unclear, (2) the feature quantities of hole edges during deformation are difficult to be measured, and so on. Therefore, extensive research is needed.

## 3. Material, and Anisotropy Coefficients in the Principal Axis Directions

### 3.1. Material

The material of this study is aluminum alloy AA6061-O extruded thin-walled tubes, which have obvious in-plane anisotropy and are widely used in aerospace, automotive, and other fields due to their excellent mechanical properties. The initial outer diameter of the tube is 60 mm and the initial thickness is 2 mm. 

### 3.2. Anisotropic Yield Criterion

The Hill48 yield criterion is used to characterize the plastic anisotropy of the material, due to this study only focusing on the in-plane anisotropic plastic flow characteristics of the tube. For the plane stress state, the Hill48 yield criterion is expressed as [34]:

(2) $$2f\left(\sigma_{ij}\right)\equiv \left(G+H\right)\sigma_{11}^{2}-2H\sigma_{11}\sigma_{22}+\left(H+F\right)\sigma_{22}^{2}+2N\sigma_{12}^{2}=1$$

where *F*, *G*, *H*, and *N* are the undetermined coefficients of Hill48 yield criterion.

Substituting the equivalent stress $\bar{\sigma }$, which is the flow stress of the axial tensile test, into Equation (2), the following equation can be obtained as:

(3) $$G+H=1/{\bar{\sigma }}^{2}$$

In order to accurately describe the in-plane anisotropic plastic flow characteristics of tubes, the in-plane anisotropy coefficients, including the axial anisotropy coefficient *r*_0_, diagonal anisotropy coefficient *r*_45_ and hoop anisotropy coefficient *r*_90_, are used to determine the undetermined coefficients of the Hill48 yield criterion, as shown in the following equations,

(4) $$r_{0}=\frac{H}{G};\;\;\;\;r_{90}=\frac{H}{F};\;\;\;\;r_{45}=\frac{N}{F+G}-\frac{1}{2}$$

According to Equations (3) and (4), the coefficients can be expressed as:

(5) $$\left\{ \begin{array}{l} G+H=1/{\bar{\sigma }}^{2}\\ 2H=\frac{2r_{0}}{{\bar{\sigma }}^{2}\left(1+r_{0}\right)}\\ H+F=\frac{r_{0}\left(1+r_{90}\right)}{{\bar{\sigma }}^{2}r_{90}\left(1+r_{0}\right)}\\ 2N=\frac{r_{0}+r_{90}}{{\bar{\sigma }}^{2}r_{90}\left(1+r_{0}\right)}\left(2r_{45}+1\right) \end{array}\right.$$

Thus, the Hill48 yield criterion can be expressed as:

(6) $$\sigma_{11}^{2}-\frac{2r_{0}}{1+r_{0}}\sigma_{11}\sigma_{22}+\frac{r_{0}\left(1+r_{90}\right)}{r_{90}\left(1+r_{0}\right)}\sigma_{22}^{2}+\frac{r_{0}+r_{90}}{r_{90}\left(1+r_{0}\right)}\left(2r_{45}+1\right)\sigma_{12}^{2}={\bar{\sigma }}^{2}$$

The anisotropic coefficients *r*_0_, *r*_90_ and hardening curve $\bar{\sigma }(\bar{\varepsilon })$ can be accurately determined by the uniaxial tensile tests and the hoop tensile test. While the anisotropic coefficient *r*_45_ will be determined by the hybrid numerical–experimental method as shown in Section 2.2.

### 3.3. Measurement of Anisotropy Coefficients in the Principal Axis Directions

**Figure 5:**
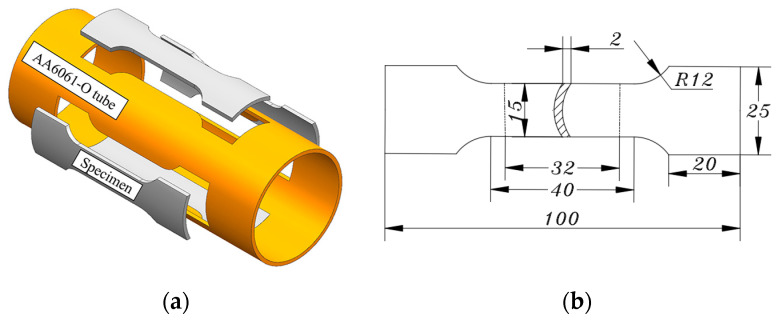
(**a**) Specimen sampling diagram, and (**b**) Specimen dimensions (unit mm).

The uniaxial tensile tests are conducted on the LE5105 100 KN electronic universal tensile testing machine to measure the axial anisotropy coefficient *r*_0_ of AA6061-O tube. Axial uniaxial tensile specimens are designed according to the Chinese Standard of GB/T 228.1-2010 [35] and cut along the axial orientation of the tube as shown in Figure 5 (every 90° in the hoop direction).

**Figure 6:**
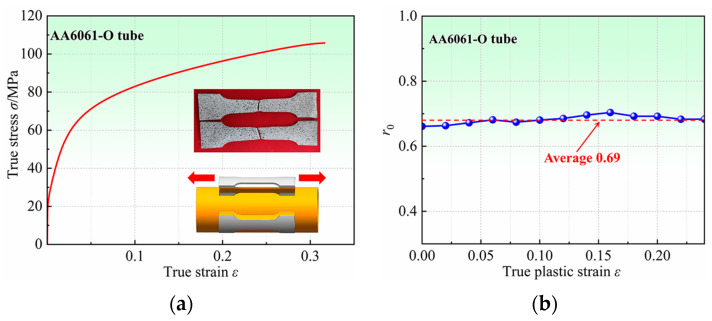
(**a**) True stress–strain curve, and (**b**) Evolution of the *r*-value (*r*_0_) with strain.

The strain is measured in real-time by a 3D digital image correlation (3D-DIC). The stress–strain curves measured using specimens cut from four locations are almost overlapping, so only one result is plotted in Figure 6a. As shown in Figure 6b, the average value of *r*_0_ is 0.69, which does not change significantly with the increase in strain.

**Figure 7:**
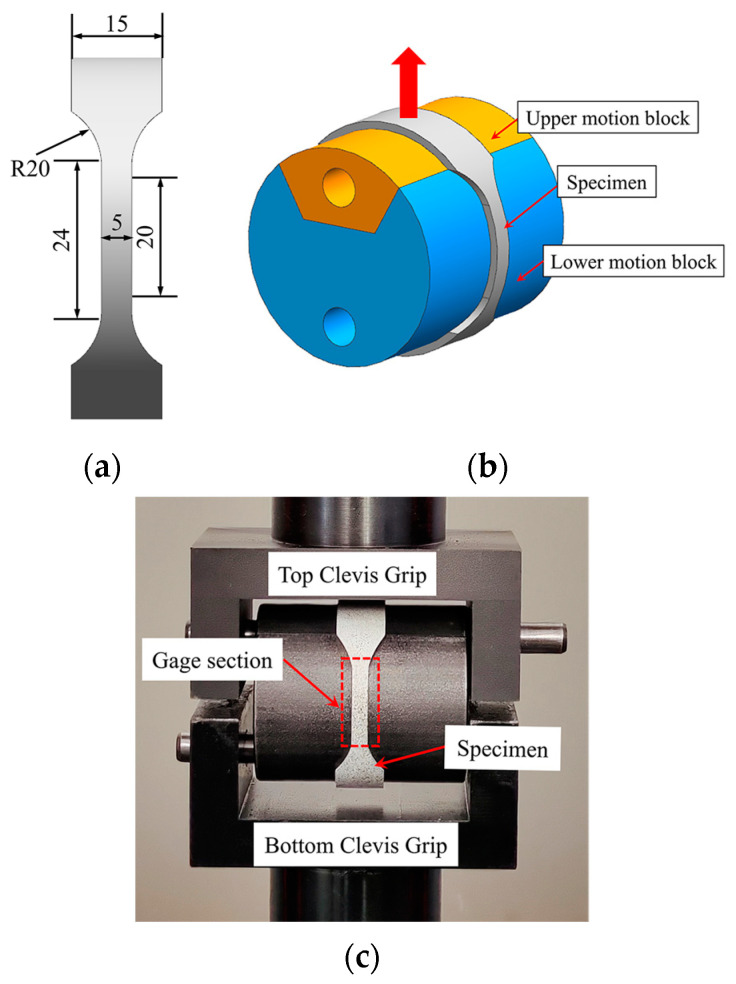
(**a**) Specimen dimensions (unit mm), (**b**) Clamps, and (**c**) Test device.

The hoop anisotropy coefficient *r*_90_ of AA6061-O tubes is measured by the hoop tensile test, and the corresponding specimen and experimental device are shown in Figure 7. The length–width ratio of the gauge section of the specimen is 4:1 and other dimensions are designed according to the Chinese standard GB/T 228.1-2010, as shown in Figure 7a.

**Figure 8:**
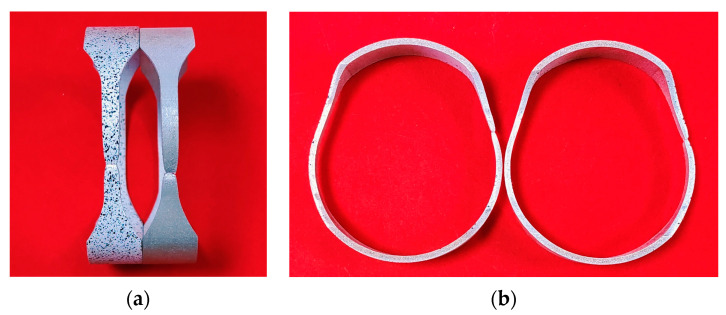
Specimens after hoop tensile tests: (**a**) Front view, and (**b**) Side view.

As shown in Figure 7b, the hoop tensile test device consists of two clevis grips and two motion blocks. The specimen is elongated and deformed by the separation of the upper and lower motion blocks. As shown in Figure 7c, compared with the traditional device with a central mandrel, the central mandrel and the lower moving block are designed as a single unit, which can reduce the influence of fixture jitter during the testing process. Keeping the gauge section at the 3 o’clock and 9 o’clock orientations of the fixture and lubricating the specimen and fixture with PVC films can minimize the influence of friction between the fixture and the specimen. The test is performed on the LE5105 100 KN electronic universal tensile testing machine with DIC for strain measurement in the gauge section. The specimens after the hoop tensile test are shown in Figure 8, and the measured *r*_90_ of AA6061-O aluminum alloy tubes is 1.05.

## 4. FE Simulation

### 4.1. FE Model

**Figure 9:**
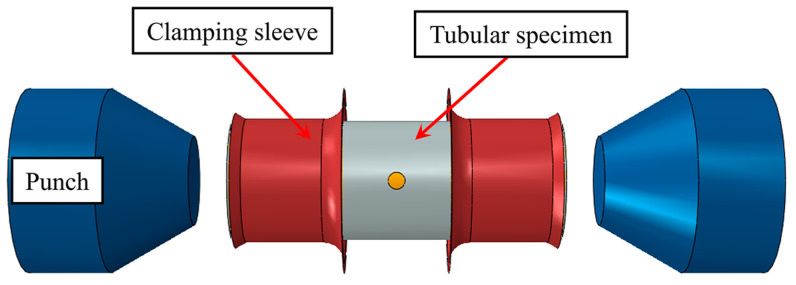
FE model for the HBT.

In this section, ABAQUS is used to simulate the HBT. As shown in Figure 9, the FE model includes the tubular specimen with a hole, the inner tube, the punches, and the clamping sleeves. The simulation process is consistent with the experiment, which includes two analysis steps: first, the displacements of the left and right punches are controlled to achieve the seal of the double-layer tube, and then the internal pressure of the double-layer tube is applied according to the actual loading path of the experiment. The punches and the clamping sleeves are set as rigid bodies. The tubular specimen with a hole and the inner tube are set to be deformable. The elastic modulus, hardening curve, and other parameters are obtained by the uniaxial tensile tests. The contact between each surface is set as face-to-face contact, and Coulomb’s law is chosen. The friction coefficient between the inner tube and the specimen is 0.02, between the inner tube and the punch is 0.15, and other contact regions are 0.17.

**Figure 10:**
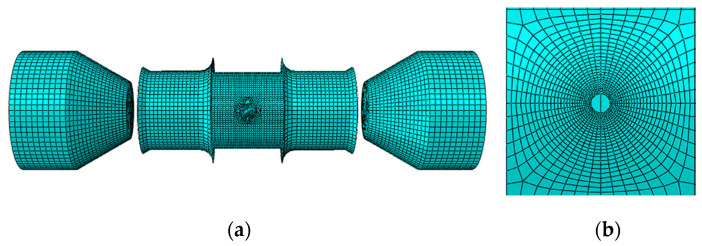
FE mesh: (**a**) the entire model, and (**b**) Local mesh near the hole.

As shown in Figure 10, the tubular specimen with a hole adopts a 4-node linear reduced integration shell element (S4R). Five integral points are set in the thickness direction, and the global element size of the tube is set to 2.0 mm. Local mesh refinement is performed around the hole, and 80 elements are evenly distributed around the hole to ensure simulation accuracy. The global size of the inner tube, the punches, and the sleeves grid are set to 3.0 mm.

### 4.2. Stress–Strain Analysis of the Hole Periphery

The stress–strain characteristics of the hole periphery in deformation are analyzed by FE simulation. When analyzing the stress and strain of each point on the edge of the hole, the coordinate system is established in the radial (r direction), tangent (ta direction), and thickness (t direction) of the point, which changes with the position of the analysis point. Each point of the hole periphery can be represented by the angle between the point and the tube axis direction.

**Figure 11:**
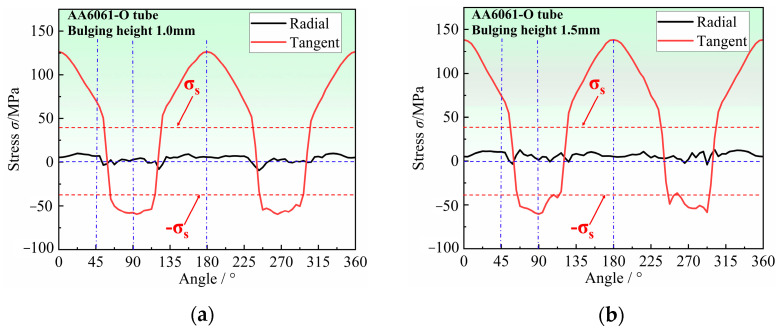

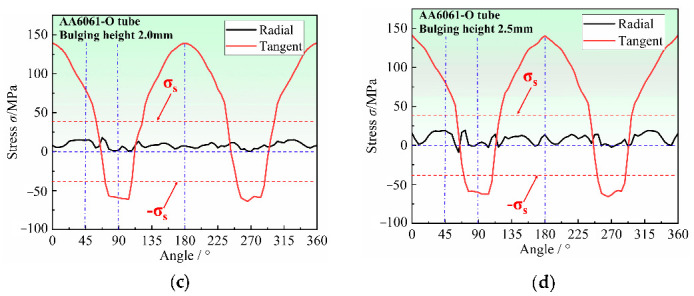
Stress distribution at the hole periphery for different bulging heights: (**a**) 1.0 mm, (**b**) 1.5 mm, (**c**) 2.0 mm, and (**d**) 2.5 mm.

Figure 11 shows the stress distribution at each point of the hole periphery under different bulging heights. The radial stress of each point at the hole periphery is almost 0 MPa at different bulging heights, while tangent stress is completely different from radial stress. From 0° to 90°, the tangential stress gradually transforms from tensile stress to compressive stress, with the maximum tensile stress at 0° and the maximum compressive stress at 90°. Therefore, it can be approximated that each point around the hole is only subjected to tangential stress and is in a uniaxial stress state. The direction of stress at each point around the hole can represent one specific orientation in the tube plane. In addition, the stress in almost all regions of the hole periphery exceeds the yield strength of the material, which indicates that plastic deformation occurs in almost all orientations around the hole, that is, all directions in the tube plane are considered to involve the deformation around the hole. Therefore, the deformation characteristics around the hole can reflect the differences in plastic flow in any orientation within the tube plane, and can be used to further determine the anisotropy coefficient of the tube. 

**Figure 12:**
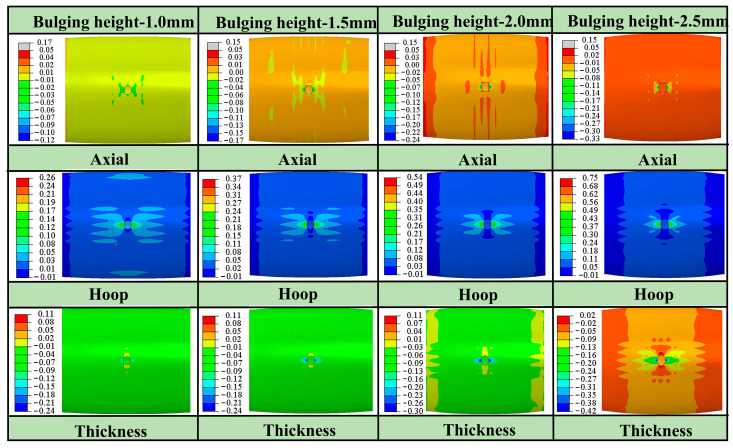
Strain contour diagrams of hole periphery with different bulging heights.

Figure 12 shows the radial, tangent, and thickness strain contour diagrams around the hole. The tangent strain at the hole periphery is the largest, with the maximum tensile strain in the 0° and 180° orientations and the maximum compressive strain in the 90° and 270° orientations, which is consistent with the variation law of tangential stress shown in Figure 11. Due to the fact that the edge of the hole is in a tangential uniaxial stress state, the position with a large positive tangent strain corresponds to a large thickness reduction, the rupture around the hole is most likely to occur at 0° and 180° positions, and the smaller the r value, the earlier the rupture occurs. The position with the negative tangential strain exhibits thickening, and the smaller the r value, the more thickening occurs. Therefore, it can be concluded that the thickness variation around the hole is closely related to the in-plane anisotropy of the tubular specimen. The thickness characteristics around the hole can reflect the differences of plastic flow in any direction within the tube plane and can be used to further determine the anisotropy coefficient of the tube. 

**Figure 13:**
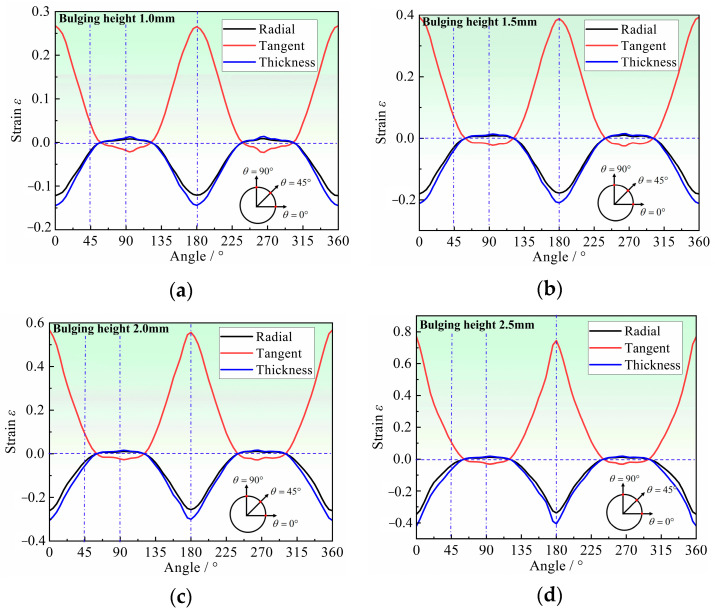
Strain distribution at the hole periphery for different bulging heights: (**a**) 1.0 mm, (**b**) 1.5 mm, (**c**) 2.0 mm, and (**d**) 2.5 mm.

Figure 13 shows the strain distribution at the hole periphery with different bulging heights. It can be seen that the radial strain and the thickness strain are different in the same position, and the difference is especially significant at the positions with large deformation, such as 0° position and 180° position. As the bulging height increases, the strain difference between the radial and thickness directions of each point at the hole periphery is more obvious. Because the stress state around the hole is uniaxial, the difference between the radial and thickness strains can directly reflect the in-plane anisotropy of the tube. Therefore, the difference between the radial strain and thickness strain around the hole can be used to determine the in-plane anisotropy of the tube. When the bulging height is constant, the greater the difference between the two, indicating that the tube has more obvious in-plane anisotropy.

Through the stress–strain analysis of the hole periphery, the deformation characteristics around the hole, such as the distribution of thickness and the difference between the radial and thickness strains, can reflect the anisotropic plastic flow characteristics of tubes, which can be used to determine the in-plane anisotropy coefficients of tubes.

### 4.3. Deformation Characteristics of the Hole Periphery

**Figure 14:**
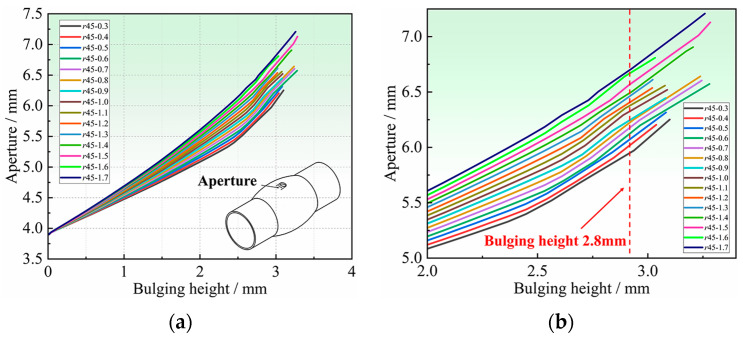
The relationship between aperture and bulging height for different *r*_45_: (**a**) Overall graph, and (**b**) Local zoomed-in graph.

**Figure 15:**
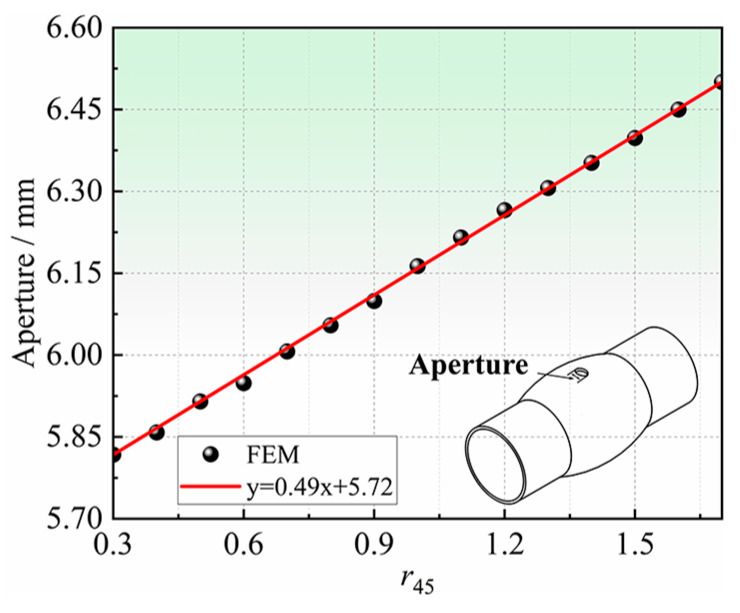
Relationship between aperture and *r*_45_ at the same bulging height.

Hole deformation characteristics mainly include aperture, thickness of hole periphery, etc. In this section, the relationship between the deformation characteristics around the hole and in-plane anisotropy coefficients, is analyzed by FE simulation. The analysis of the deformation characteristics at the hole periphery, which are highly sensitive to the anisotropy coefficients, is important for determining the anisotropy coefficients in the non-principal axis direction of the tube. Taking the Hill48 yield criterion as an example, the undetermined anisotropy coefficient is *r*_45_. The influence of *r*_45_ on the deformation characteristics of the hole periphery will be analyzed. The *r*_45_ is taken every 0.1 in the range of 0.30–1.70 for the simulation analysis. Figure 14 shows the relationship between the apertures and the bulging height with different *r*_45_. It can be seen that different *r*_45_ have obvious regularity on the influence of the apertures after bulging. The larger the *r*_45_, the larger the aperture at the same bulging height, and the aperture difference will increase with the increase in bulging height. The apertures for different *r*_45_ were extracted when the bulging height was 2.80 mm, as shown in Figure 15. At the same bulging height, the larger the *r*_45_, the larger the aperture after bulging. There is a one-to-one correspondence between *r*_45_ and the apertures, which indicates that it is feasible to determine *r*_45_ inversely by the apertures.

**Figure 16:**
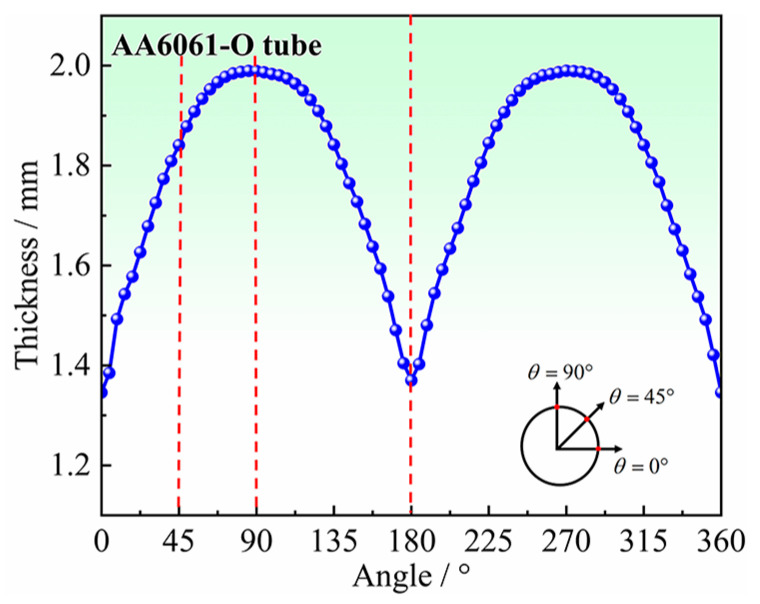
Thickness of hole periphery at 2.5 mm bulging height.

At 2.5 mm bulging height, the thickness around the hole was extracted, as shown in Figure 16. The thickness reduction is the largest at the orientations of 0° and 180°, reaching about 0.6 mm, while there is almost no thinning in the direction of 90° and 270° (note that initial thickness is 2 mm), and even a little thickening, which is consistent with the results of Section 4.2 hole periphery strain analysis.

**Figure 17:**
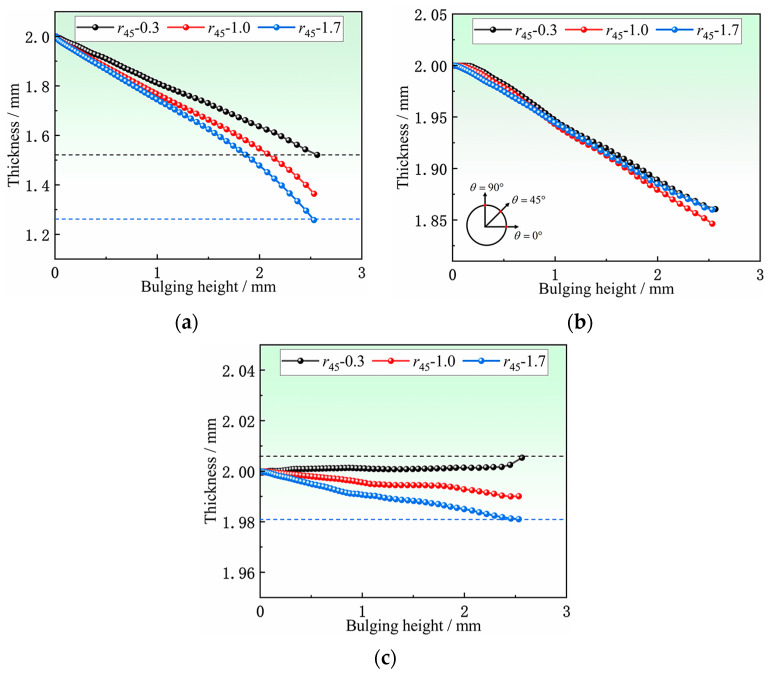
Variation of thickness with bulging height at different locations of hole periphery: (**a**) 0°, (**b**) 45°, and (**c**) 90°.

As shown in Figure 17, the variations of thickness with bulging height in three orientations of 0°, 45°, and 90° under different *r*_45_ are analyzed. The thicknesses of 0°and 90° are more sensitive to the changes of *r*_45_ than that of 45°. The thickness of 0° is the most sensitive to *r*_45_. When *r*_45_ changes from 0.3 to 1.7 with the bulging height of 2.5 mm, the difference in thickness thinning at the position of 0° is 0.27 mm, and the difference rate reaches 13.5%. This is because throughout the entire HBT, the stresses near 45° and 90° positions are always very small which lead to a small deformation amount at both locations, so the differences in deformation that could be reflected are very small. However, the stress near 0° is always the maximum stress around the hole, and the deformation amount is large, which naturally better reflected the difference in deformation, as shown in Figure 11. Fortunately, the wall thickness at the 0° position can well reflect the changes in r45, so the wall thickness, especially at the 0° position, can be used to determine the anisotropy coefficient of the thin-walled tubular specimens in reverse.

In summary, both the aperture of the hole and the thickness around the hole (especially thickness in the 0° orientation) can reflect the in-plane anisotropy of tubes, and have a significant correlation with the undetermined anisotropy coefficient *r*_45_. Considering that the aperture is easy to measure accurately, the aperture is chosen as the iterative metric of the hole deformation used to determine the anisotropy coefficients in the non-principal axis directions in Section 2.2.

### 4.4. Influencing Factors of Hole Deformation

The strength relationship between the inner tube and the tubular specimen, the initial aperture, and the friction coefficient between the inner and outer tube on the hole deformation were analyzed by FE simulations. The optimal experimental conditions are determined to make the hole deformation characteristics significantly reflect the in-plane anisotropy of tubes and reduce the influence of external factors on the experimental results.

#### 4.4.1. Strength Relationship between the Inner and Outer Tube

**Figure 18:**
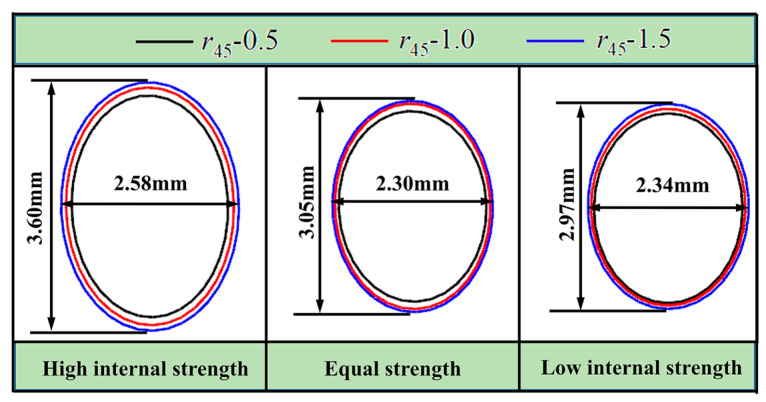
Effect of different inner and outer tube strength relationships on the shape of the hole after deformation.

The inner and outer tube strength-matching relationship can be categorized as “high internal strength” (HIS), “equal strength” (ES), and “low internal strength” (LIS). As shown in Table 1, the ultimate bulging height is the maximum expansion height of the hole before rupturing at *r*_45_ = 1.5, the aperture is the major axis length of the hole under the ultimate bulging height in this study, and the deformation difference is the difference between the long axis after experiment and the initial aperture divided by the initial aperture. From Table 1, it can be seen that the ultimate bulging height, aperture, and deformation difference degree corresponding to HIS are the largest, which will be more beneficial to reflect the deformation difference caused by different *r*_45_ values. As shown in Figure 18, the overall hole size of HIS is the largest at the ultimate bulging height, and the difference in hole size between different anisotropic coefficients is the most significant. Thus, it is recommended to choose HIS for the HB experiments, and a 304 stainless steel tube was selected as the inner tube in this paper as it has higher strength than AA6061-O tube. In addition, 304 stainless steel tubes are generally isotropic, so it is modeled with the Mises yield criterion in the simulation.

#### 4.4.2. Effect of Initial Aperture

The hole bulging tests with initial apertures of 2 mm, 3 mm, 4 mm, and 5 mm were simulated and analyzed, respectively, and the results are shown in Table 2. The ultimate bulging height in the table is the maximum bulging height before the hole ruptures when *r*_45_ = 1.5. The aperture and deformation difference degree are consistent with the definition in Section 4.4.1. A small initial aperture corresponds to a large ultimate impacting height and a large deformation difference degree.

**Figure 19:**
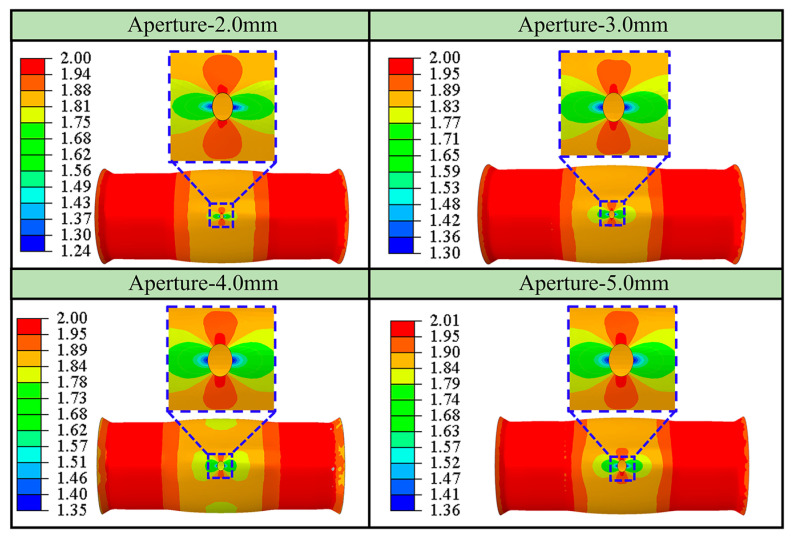
Thickness distribution at ultimate bulging height for different initial aperture.

Figure 19 shows the thickness distribution at the hole periphery with different initial apertures at the ultimate bulging height. The results show that the thickness distribution is not very different when the initial aperture changes. The thickness at a position of 0° is the smallest, and the thickness at a position of 90° is the largest.

**Figure 20:**
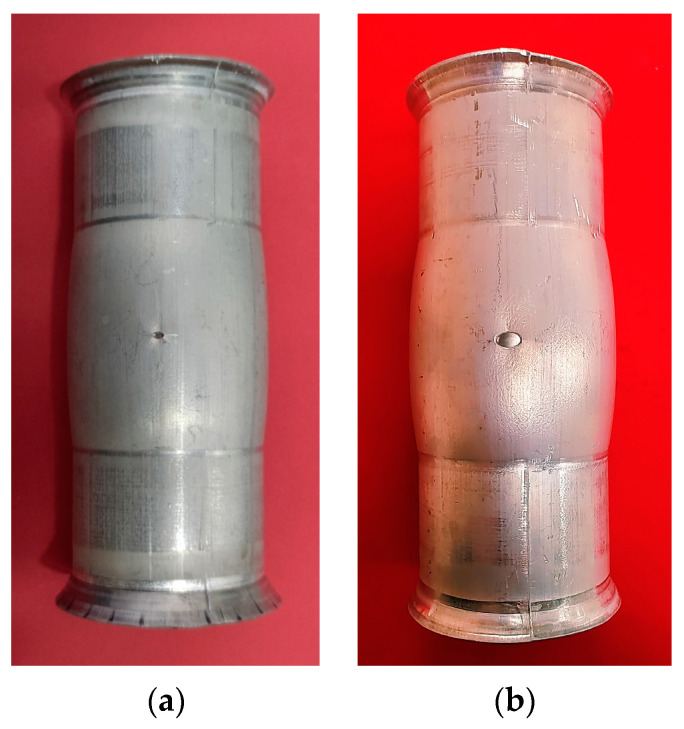
Tubular specimens after HB experiments with initial apertures of: (**a**) 2 mm, and (**b**) 4 mm.

From the above results, the smaller the initial aperture is, the larger the ultimate bulging height is, and the larger the hole deformation is. However, a small initial aperture will increase the difficulty of measurement, resulting in increased measurement error. Although the ultimate bulging height of the large aperture is smaller, the size of the aperture changes more greatly, which is easier to be measured. To balance this problem, preliminary HB experiments were carried out on 2 mm and 4 mm apertures, as shown in Figure 20. The experimental results are basically consistent with the simulation results. The ultimate bulging height of the tube with 2 mm aperture is 3.34 mm, while at 4 mm aperture it is 3.10 mm. However, when measuring the aperture after deformation, the 2 mm aperture tube is difficult to be measured and the measurement error is large, while more accurate measurement values can be obtained for the 4 mm aperture tube. Therefore, to obtain more accurate experimental results, tubes with 4 mm initial aperture are chosen to conduct HB experiments.

#### 4.4.3. Friction between Inner and Outer Tubes

For different *r*_45_, the friction coefficients between the inner and outer tubes are set to 0.1, 0.2, and 0.3 for FE simulations, and the apertures at the bulging height of 2.75 mm are extracted, as shown in Table 3. It can be seen that the difference of apertures at the same bulging height is very small when *r*_45_ is the same and the friction coefficient increases from 0.1 to 0.3, with the maximum difference not exceeding 1%. Therefore, it can be concluded that the friction coefficient has little effect on the deformation of the hole and is not an important factor. During the HB experiments in this paper, the inner tube is wrapped by PVC films to fully reduce the friction between the two tubes.

## 5. Hole Bulging Experiment

### 5.1. Experimental Process

Before the HB experiments, the AA6061 tubes were annealed by holding the temperature at 405 °C for 3 h, cooled to 260 °C with the furnace, and then air-cooled. The total length of the tubular specimen with a hole and the length of the bulging zone used in this study are 160 mm and 70 mm, respectively. A hole with a diameter of 4 mm was drilled in the middle of the tubular specimen. The inner tube is a 304 stainless steel tube, the initial diameter, thickness, and total length of which are 55 mm, 1 mm, and 160 mm, respectively.

**Figure 21:**
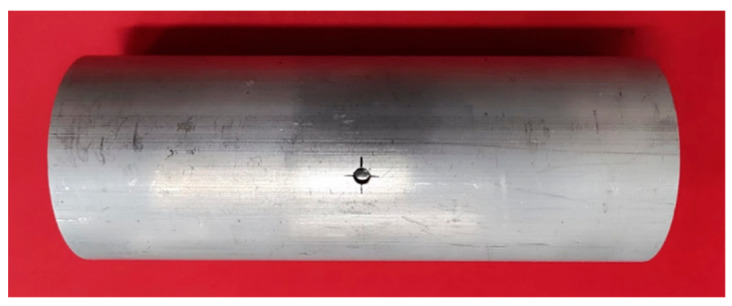
Schematic diagram of the tubular specimen with a hole before experiment.

The measurement points are marked at the hole periphery as shown in Figure 21. The surface of the inner tube is wrapped around by PVC films. The inner tube is placed inside the tubular specimen with a hole. Then the double-layer tube is installed on the equipment shown in Figure 4. The double-layer tube is sealed and loaded according to the preset pressure loading path to bulge the tubular specimen with holes. After the experiments, the apertures, the thicknesses at the hole periphery, and the bulging zone contours were measured.

### 5.2. Experimental Results

**Figure 22:**
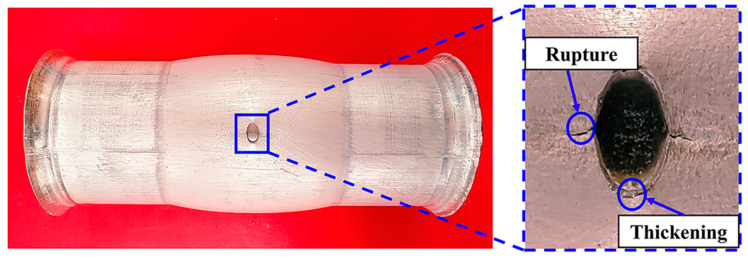
Experimental results of 3.31 mm bulging height.

Figure 22 is the tubular specimen with an initial hole diameter 4 mm after HB experiment with 3.31 mm bulging height. The hole ruptures at the positions of 0° and 180°, and local thickening occurs at the positions of 90° and 270°, which are consistent with the simulation results.

**Figure 23:**
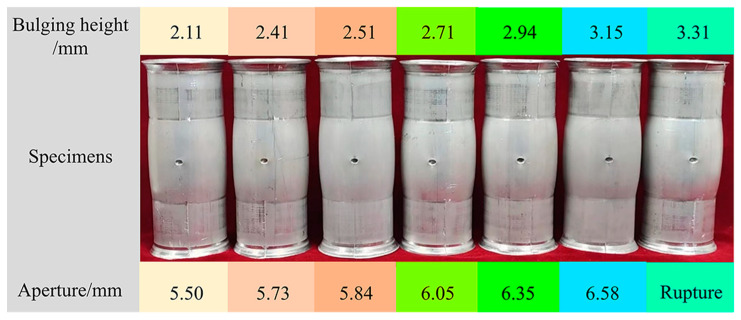
Test results of the HB experiments.

Seven groups of experiments under different bulging heights are conducted on the same batch of tubes. During the HB experiments, the bulging height is measured in real-time by a laser displacement sensor. The results are shown in Table 4 and Figure 23.

## 6. Results and Verification

### 6.1. Results of r-Value

**Figure 24:**
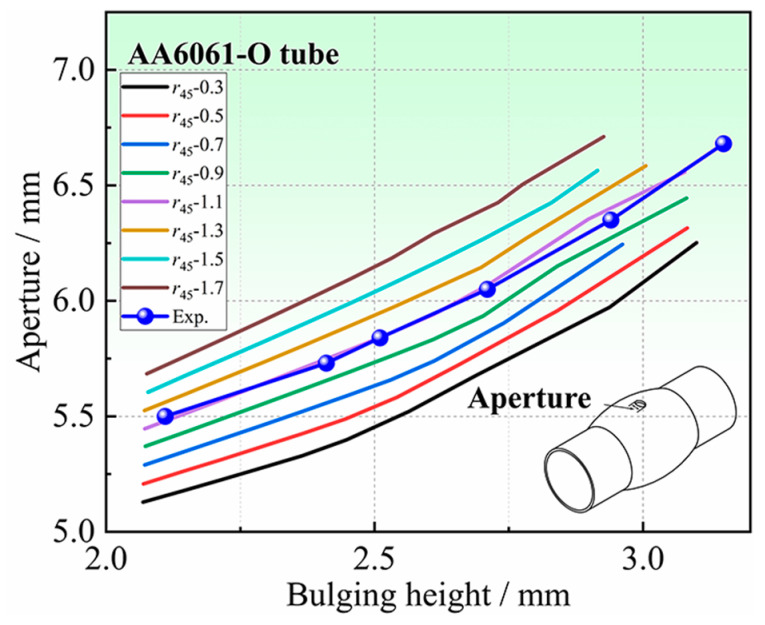
The bulging height–aperture curve of tubular specimen with a hole.

When determining anisotropy coefficients in the non-principal axis directions using the method in Section 2.2, it is necessary to determine the iterative range of the undetermined anisotropy coefficients. In this study, the iterative range of the anisotropy coefficient *r*_45_ is determined by comparing the results of HB experiments and FE simulations. As shown in Figure 24, the bulging height–aperture curve obtained by the HB experiments is located between the FE simulation results of *r*_45_ = 0.9 and *r*_45_ = 1.1. Therefore, it can be concluded that the actual *r*_45_ of the tube is between 0.9 and 1.1. The iterative range of the *r*_45_ is determined to be [0.9, 1.1].

**Figure 25:**
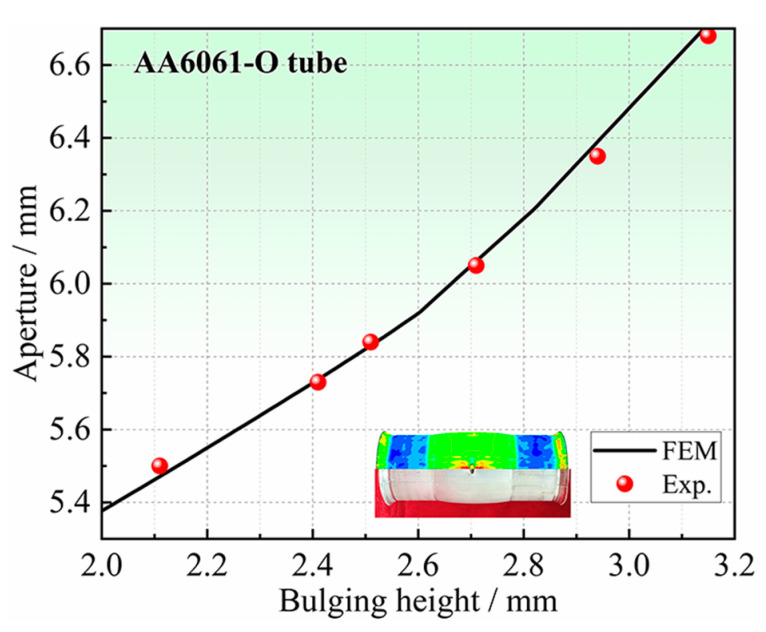
Comparison of aperture between HB experiments and simulation results for *r*_45_ = 1.06.

Through the identification process of anisotropic coefficients proposed in Section 2.2, the RMSE (see Equation (1)) threshold is $\Delta_{\eta }=0.5\%$, and the iteration results are shown in Table 5. Finally, *r*_45_ = 1.06 is determined, and the RMSE between the results of experiments, and FE simulations of *r*_45_ = 1.06 is 0.47%. The difference in the bulging height–aperture curve is also the smallest, as shown in Figure 25, and the maximum error is 0.51%, which indicates that the proposed method is reasonable.

### 6.2. Verification

Other characteristics of the tubular specimen with a hole after the experiments can be used to verify the accuracy of the determined anisotropy coefficients, such as the thickness around the hole and the profile of the bulging zone. The *r*_45_ determined in Section 6.1 is substituted into the simulation model for FE simulation, and the simulation results of the hole periphery thickness and the contour of the bulging zone are compared with the experimental results.

**Figure 26:**
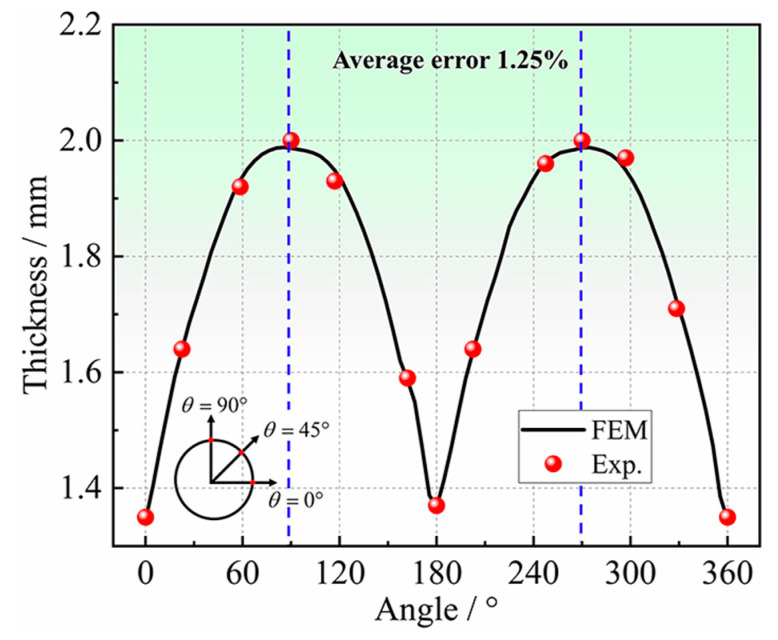
Comparison of thicknesses at the hole periphery from experiment and simulation with *r*_45_ = 1.06.

Figure 26 shows the results of the thickness around the hole at the bulging height of 2.50 mm, obtained by the experiment, and FE simulation of *r*_45_ = 1.06. The thicknesses obtained by FE simulations are in good consistency with the experimental results, and the average error is only 1.25%. From the experimental results, the thickness reduction is the largest at the position of 0°, which is 0.65 mm. The thickness reduction decreases gradually from 0° to 90°, and there is a very small thickness thickening at the position of 90°.

**Figure 27:**
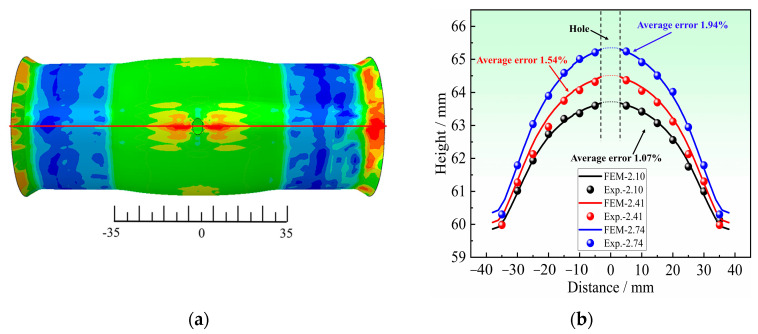
Comparison of experimental and simulated contour of the bulging zone for *r*_45_ = 1.06: (**a**) Distribution of sampling points, and (**b**) Comparison of the contour of the bulging zone.

As shown in Figure 27a, the whole bulging zone is divided into 15 sampling points. The profiles of the bulging zone obtained by the HB experiments at the bulging heights of 2.10 mm, 2.41 mm, and 2.74 mm are compared with the corresponding results of FE simulation, as shown in Figure 27b. The comparison results indicate that they are in good consistency, and the average error of the contour is the largest at the bulging height of 2.74 mm, which is 1.94%.

From the comparative results of the thickness around the hole and the profile of the bulging zone, the hole deformation characteristics of the HBT can reflect the anisotropic plastic flow characteristics of tubes. It is feasible to determine the non-principal axis directions anisotropy coefficient of tubes through the hybrid numerical–experimental method based on the HBT, and the anisotropy coefficients obtained can guarantee high accuracy.

### 6.3. Results of AA6061-O in-Plane Anisotropy

The Hill48 plastic potential equation of AA6061-O extruded aluminum alloy tubes can be obtained by substituting *r*_0_ = 0.67, *r*_45_ = 1.06, and *r*_90_ = 1.05 determined in this study into Equation (6).

(7) $$\sigma_{11}^{2}-0.80\times \sigma_{11}\sigma_{22}+0.78\times \sigma_{22}^{2}+3.09\times \sigma_{12}^{2}={\bar{\sigma }}^{2}$$

According to the Drucker flow rule, the anisotropy coefficients of AA6061-O extruded aluminum alloy tubes in any direction *θ* is:

(8) $$r_{\theta }=\frac{1.45\times \cos^{4}\theta +0.95\times \sin^{4}\theta +\cos^{2}2\theta +1.87\times \sin^{2}2\theta }{1.45\times \cos^{2}\theta +0.95\times \sin^{2}\theta }-1$$

**Figure 28:**
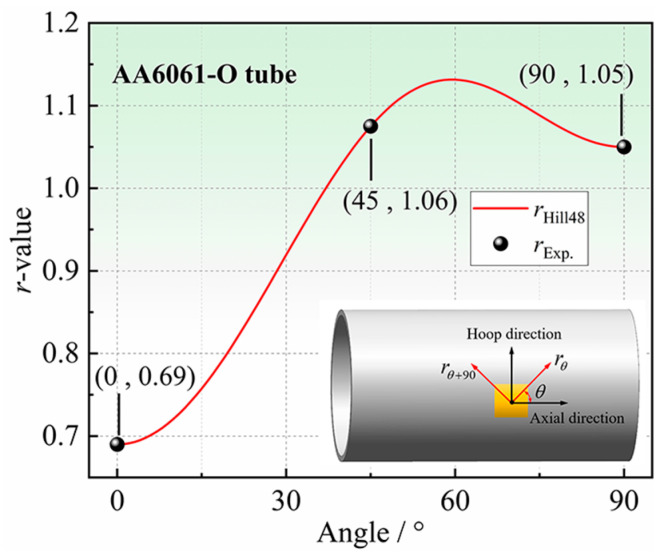
AA6061-O extruded aluminum alloy tubes in-plane anisotropy coefficients.

The anisotropy coefficients of AA6061-O extruded aluminum alloy tubes in any non-principal axis directions are given for the first time, as shown in Figure 28. The in-plane anisotropy coefficients of AA6061-O extruded aluminum alloy tubes increase first and then decrease from 0° to 90°, and reach the maximum value of 1.13 in the orientation of 60°.

## 7. Conclusions

In this study, in order to determine the anisotropic coefficients in non-principal axis directions of thin-walled tubes, a new experiment was proposed, which was called the hole bulging test (abbreviated as HBT). The specimen of HBT is a tube with a hole in the axial center. The specimen is inserted into a tube without a hole. The hole on the specimen expands simultaneously as the double-layer tube undergoes free bulging under internal pressure. Furthermore, based on the information obtained from the proposed experimental method, a hybrid numerical–experimental method was used to identify the anisotropic coefficients of tubes. The thickness, stress, and strain state around the hole, the hole shape, as well as the factors that affect the hole deformation were analyzed through FE simulations and the aperture reflecting the hole shape was determined as the iterative metric of the hybrid numerical–experimental method. Uniaxial tensile tests and hoop tensile tests were performed to determine the tubular materials’ yield stresses, hardening curve, and anisotropy coefficients with respect to axial and hoop directions. The HB experiments and FE simulations of the aluminum alloy AA6061-O extruded tube were performed, and the undetermined anisotropic coefficient in the plastic constitutive model was identified by the hybrid numerical–experimental method and the accuracy of it was further verified. Finally, the in-plane anisotropic coefficients in any direction of AA6061-O extruded tube were determined through the plastic flow rule. The main conclusions are as follows:

1. In the HBT, the stress state around the hole is uniaxial, and the hole deformation is a comprehensive result of the deformations of various points around the hole, which can reflect the in-plane anisotropic plastic flow characteristics of the tube.
2. The aperture of the hole and thickness around the hole after deformation vary significantly with the variation of anisotropy coefficients in the non-principal axis directions of thin-walled tubes, and can be used to determine them.
3. For the HBT, a higher strength of the inner tube than the tubular specimen, and a smaller initial diameter of the circular hole are recommended. The friction coefficient between the double-layer tube has little effect on the hole deformation.
4. Compared with the experimental results, both the average errors of the thickness around the hole and the profile of the bulging zone obtained from the final iterative simulation analysis do not exceed 2%, verifying the feasibility of the proposed method in this paper.
5. The aluminum alloy AA6061-O extruded tube exhibits significant in-plane anisotropic plastic flow characteristics, and its in-plane anisotropic coefficients in any direction are given for the first time, which increase first and then decrease from 0° to 90°, reaching a maximum value of 1.13 at 60°, a minimum value of 0.69 at 0°.

The above conclusions reveal that the HBT is a promising test for accurate identification of the in-plane anisotropic coefficients of the non-principal axial directions of thin-walled tubes. This study only investigates the feasibility and application effect of the HBT. In future research, the feasibility of directly measuring the in-plane anisotropic coefficients of the non-principal axial directions of thin-walled tubes through the HBT will be explored.

## Figures and Tables

**Table 1:** Effect of strength relationship between inner and outer tube on hole deformation.

Strength Relationship	HIS	ES	LIS
Ultimate bulging height/mm	3.20	3.11	2.95
Aperture/mm	3.60	3.05	2.97
Deformation difference degree/%	80.0	52.5	48.5

**Table 2:** Simulation results for different initial apertures.

Initial Aperture/mm	2.0	3.0	4.0	5.0
Ultimate bulging height/mm	3.25	3.18	3.10	2.89
Aperture/mm	3.66	5.30	6.75	7.80
Deformation difference degree/%	83.0	76.7	68.8	56.0

**Table 3:** Aperture after deformation with different friction coefficients.

Friction Coefficient	0.1	0.2	0.3
*r*_45_ = 0.5	5.58 mm	5.58 mm	5.60 mm
*r*_45_ = 1.0	5.82 mm	5.80 mm	5.83 mm
*r*_45_ = 1.5	6.08 mm	6.01 mm	6.03 mm

**Table 4:** Test results of the HBT at different bulging heights.

Bulging Height/mm	Internal Pressure/MPa	Aperture/mm
2.11	28.07	5.50
2.41	29.34	5.73
2.51	29.68	5.84
2.71	30.02	6.05
2.94	30.32	6.35
3.15	30.51	6.58
3.31	30.70	Rupture

**Table 5:** Iterative results.

Iterative Methods	Number of Iterations	Iteration Step Size	*r*_45_	$\mathrm{RMSE}\;\bar{\eta }$
Bisection method			0.9	1.47%
			1.1	0.53%
	1	0.1	1.0	0.68%
	2	0.05	1.05	0.53%
Fixed step method	3-1	0.01	1.06	0.47%
	3-2	0.01	1.07	0.51%
	3-3	0.01	1.08	0.60%
	3-4	0.01	1.09	0.64%

## Data Availability

Not applicable.

## Nomenclature

HBT	Hole bulging test, a new experiment proposed in this paper	HET	hole expansion test of sheet metal
$r_{\theta }$	Anisotropy coefficient of the tube, θ is the direction relative to the axial direction	$R_{i}^{exp}$	the values of the iterative metric in the results of experiment
$R_{i}^{fem}$	the values of the iterative metric in the results of simulation	$\Delta_{\eta }$	the iteration step
$\bar{\sigma }$	the equivalent stress, which is the yield stress of the axial tensile test	$\sigma_{ij}$	Stress tensor
HIS	High internal strength, that is, the strength of the inner tube is higher than the strength of the tubular specimen	ES	Equal strength, that is the strength of the inner tube is equal to the strength of the tubular specimen
LIS	Low internal strength, that is, the strength of the inner tube is lower than the strength of the tubular specimen		
